# 9-Genes Reinforce the Phylogeny of Holometabola and Yield Alternate Views on the Phylogenetic Placement of Strepsiptera

**DOI:** 10.1371/journal.pone.0011887

**Published:** 2010-07-29

**Authors:** Duane D. McKenna, Brian D. Farrell

**Affiliations:** 1 Department of Biological Sciences, University of Memphis, Memphis, Tennessee, United States of America; 2 Museum of Comparative Zoology, Harvard University, Cambridge, Massachusetts, United States of America; University of California San Diego, United States of America

## Abstract

**Background:**

The extraordinary morphology, reproductive and developmental biology, and behavioral ecology of twisted wing parasites (order Strepsiptera) have puzzled biologists for centuries. Even today, the phylogenetic position of these enigmatic “insects from outer space” [1] remains uncertain and contentious. Recent authors have argued for the placement of Strepsiptera within or as a close relative of beetles (order Coleoptera), as sister group of flies (order Diptera), or even outside of Holometabola.

**Methodology/Principal Findings:**

Here, we combine data from several recent studies with new data (for a total of 9 nuclear genes and ∼13 kb of aligned data for 34 taxa), to help clarify the phylogenetic placement of Strepsiptera. Our results unequivocally support the monophyly of Neuropteroidea ( = Neuropterida + Coleoptera) + Strepsiptera, but recover Strepsiptera either derived from within polyphagan beetles (order Coleoptera), or in a position sister to Neuropterida. All other supra-ordinal- and ordinal-level relationships recovered with strong nodal support were consistent with most other recent studies.

**Conclusions/Significance:**

These results, coupled with the recent proposed placement of Strepsiptera sister to Coleoptera, suggest that while the phylogenetic neighborhood of Strepsiptera has been identified, unequivocal placement to a specific branch within Neuropteroidea will require additional study.

## Introduction

**Figure 1:**
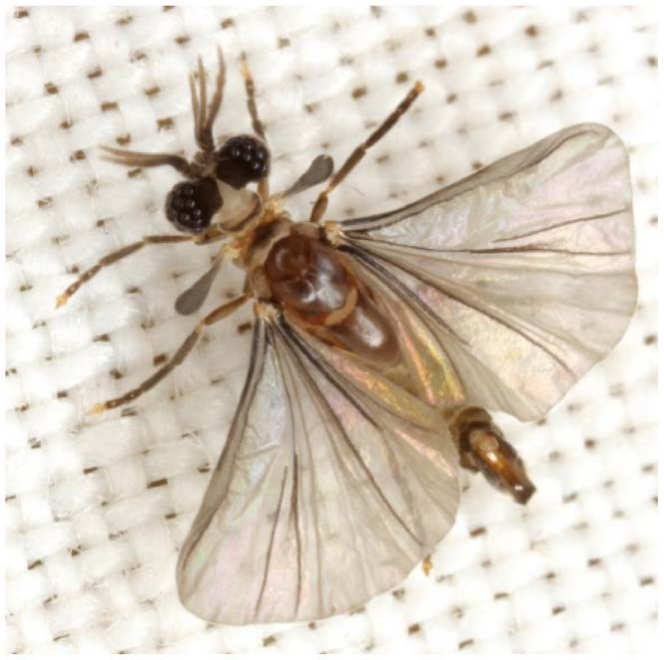
Dorsal view of adult male Corioxenidae (Strepsiptera) (photo copyright Mike Quinn, TexasEnto.net).

Twisted wing parasites (order Strepsiptera; >500 species) are cosmopolitan obligate endoparasitoids collectively using insects in 7 orders (Blattodea, Diptera, Hemiptera, Hymenoptera (particularly Aculeata), Mantodea, Saltatoria and Zygentoma) and at least 33 families as hosts [2]. Strepsiptera is comprised of 8 families in the suborder Stylopidia, and 3 extinct and 2 extant non-stylopidian families [3]–[6]. Strepsiptera have two morphologically distinct immature stages, a host-seeking 1^st^ instar “triungulin” larva adapted to reach its host by phoresy, and 3 subsequent endoparasitic instars [7], [8]. Strepsiptera parasitize their hosts at the host larval/nymphal stage and continue their development into the host's adult stage [8]. Adult Strepsiptera exhibit extreme sexual dimorphism. Females are wingless, eyeless, larviform and usually endoparasitic. Only the anterior part of the body is externally exposed, the rest is concealed in the abdomen of the host (except in the family Mengenillidae in which females are free-living and partly leave their larval exuviae, the proposed pleisiomorphic condition [3], [8], [9]). Male Strepsiptera are short-lived (3–6 hours) and free-living. They have flabellate antennae, large raspberry-like eyes likened to those of trilobites [4], [10]–[12], reduced forewings and large fan-shaped hindwings (Fig. 1). Females are fertilized by haemocoelic insemination and reproduce by haemocoelous vivipary [7], [8].

The phylogenetic affinity of Strepsiptera remains uncertain despite more than two centuries of study [e.g., 4,10,13–30]. Recent authors have argued for the placement of Strepsiptera: (a) in the beetle suborder Polyphaga [22], [31] (note, these authors did not use Hennigian or cladistic methods), (b) as a close relative of beetles [4], [10], [23], [28]–[30], [32], [33], or (c) as a sister group to true flies (order Diptera), united in a clade called “Halteria” [24], [25], [34]–[37]. It has even been suggested that Strepsiptera may be the sistergroup of the remaining Holometabola (also known as Endopterygota) [38], [39]. The natural phylogenetic placement of Strepsiptera has thus aptly been characterized as one of the most enigmatic in insect systematics (the “Strepsiptera problem” of Kristensen [38]).

While the hypothesis that Strepsiptera are a sister group to true flies (Diptera) is based on both morphological and molecular data [e.g., 34,36], both lines of evidence remain highly controversial [40]–[46]. Early reports, based on a phylogeny inferred from parsimony analysis of 18S rDNA sequence data, suggested that a homeotic mutation could account for the presence of wings on different thoracic segments in Strepsiptera and Diptera [34] (Dipteran halteres are found on the 3rd thoracic segment (metathorax) in the place of hind wings, while the “halteres” of male Strepsiptera are on the 2nd thoracic segment (mesothorax) in place of forewings). However, no genetic evidence for such a mutation has been found [47], and most of the morphological characters shared by Antliophora and Mecopterida, in which Halteria would reside, are inapplicable or absent in Strepsiptera [28], [29], [37], [44], [46], [48], [49]. Huelsenbeck [41] showed that maximum likelihood analysis of the 18S rDNA data set of Carmean and Crespi [40] recovers Strepsiptera and Coleoptera as close relatives, and suggested that long-branch attraction (LBA) — the spurious grouping of rapidly evolving sequences due to non-inherited similarity of accumulated mutations [50] — accounts for the placement of Strepsiptera sister to Diptera under parsimony inference. Subsequent studies of an engrailed homeobox intron [47] and of ecdysone receptor and ultraspiracle proteins [51], [52] recovered no evidence for a close relationship between Diptera and Strepsiptera. Casting further doubt on the Halteria hypothesis, a recent phylogenetic study of Holometabola using six single-copy nuclear protein-coding genes [28], [29] recovered Strepsiptera as a sister group to Coleoptera, and showed that this relationship was not an artifact of LBA or other systematic biases. Nonetheless, because Wiegmann et al. [28], [29] included exemplars from only one of the four suborders of Coleoptera (the “advanced” suborder Polyphaga [53]–[55]), it remains unclear whether their results are due to a close relationship between Strepsiptera and Coleoptera [4], [10], [23], [28], [29], [32], or whether Strepsiptera are derived from within Coleoptera [18], [20]–[22]. Another recent study of holometabolan relationships using DNA sequence data also recovered Strepsiptera as close relatives of beetles [30]; however, their taxon sample lacked representatives from the primitive beetle suborder Archostemata, the supra-ordinal group Neuropterida (orders Megaloptera, Neuroptera and Raphidioptera), and the order Mecoptera.

To help clarify the phylogenetic affinity of Strepsiptera, we analyzed a supermatrix of DNA sequence data comprised of 9 nuclear genes and more than 13 kb of aligned data from 34 insects including representatives of all 11 holometabolous insect orders and two hemimetabolous insect outgroups, and 8 Coleoptera representing all four extant suborders. This is the largest data set assembled to date that includes a comprehensive sample of beetle suborders and holometabolous insect orders for investigation of the phylogenetic placement of Strepsiptera.

## Materials and Methods

### Taxon sampling, DNA isolation, amplification & sequencing

We prepared a DNA sequence data set comprised of approximately 13 kb of aligned data from the 7 single-copy nuclear protein-coding genes: elongation factor-1α (EF-1α), alanyl-tRNA synthetase (AATS), carbamoylphosphate synthase domain (CAD), 6-phosphogluconate dehydrogenase (PGD), sans fille (SNF), triosephosphate isomerase (TPI), and RNA polymerase II (RNA Pol II), and two nuclear ribosomal genes: 28S and 18S. Our taxon sample was comprised of 34 insects, including 32 exemplars representing all orders of holometabolous insects [Coleoptera (8), Diptera (4), Hymenoptera (3), Lepidoptera (2), Mecoptera (5), Megaloptera (1), Neuroptera (3), Raphidioptera (1), Siphonaptera (2), Strepsiptera (2), and Trichoptera (1)], and two hemimetabolous insect outgroups (from the orders Dictyoptera & Thysanoptera) (Table S1).

All 29 taxa and 6 genes from Wiegmann et al. [29] were included in our study, except for *Boreus* sp. (Boreidae), which was excluded to eliminate generic redundancy [*Boreus brumalis* (Boreidae) was retained]. To these, we added data from 6 Coleoptera, including representatives of all four extant suborders, for a total of 7 families [56] and 8 species. We increased the gene sample of Wiegmann et al. [29] from six to nine and nearly doubled the number of nucleotide positions by adding DNA sequence data from EF-1α, 18S and 28S. Most of the added sequences (from EF-1α, CAD, RNA Pol II, 18S and 28S) were obtained from GenBank, and had been previously published, e.g., by Wild and Maddison [57] or Whiting [24], [58], but several EF-1α, 18S and 28S sequences, particularly for Coleoptera, were generated *de novo* for this study (Tables S1, S2).

On account of our desire to reduce the amount of missing data in the matrix, most taxa were ultimately represented in the matrix by family- or genus-level chimeras of DNA sequence data (with entire gene fragments contributed by each constituent taxon) (Table S1). We employed three higher-level (supra-familial) chimeras: “Megaloptera”, a chimera of *Nigronia* (Corydalidae; CAD, PGD, TPI, 18S) and *Sialis* (Sialidae; EF1a, 28S); “Halictophagidae/Myrmecolacidae”, a chimera of Halictophagidae Gen. sp. (AATS, CAD, PGD, TPI, RNA Pol II) and *Caenocholax* sp. (18S, 28S, EF-1α) - both derived relative to the other strepsipteran [*Mengenilla* (Mengenillidae)] included in this study [9]; and “Dictyoptera”, a chimera of *Blatella germanica* (AATS, CAD, PGD, SNF, TPI, RNA Pol II, 18S, 28S) and *Periplaneta americana* (EF-1α).

### Sequence alignment

We used the 5736 bp published alignment and sequences of Wiegmann et al. [29; TreeBase accession number M4658] for AATS (915 bp), CAD (2058), TPI (498 bp), SNF (561 bp), PGD (804 bp), and RNA POL II (900 bp). Supplementary sequences for CAD and RNA POL II from GenBank and unpublished sequences for these genes (courtesy, D. Maddison) from *Hydroscapha natans* (Coleoptera: Myxophaga) were manually and unambiguously aligned to this matrix. Alignment of 18S and 28S rDNA was implemented in the program MAFFT 6 using the E-INS-i method [59]. Extensive regions of ambiguous alignment mostly corresponding to known expansion regions remained in 18S and 28S after alignment. We used Gblocks 0.91b [60], [61] to identify and eliminate these ambiguously aligned positions (with the following options: smaller final blocks, gap positions within the final blocks, and less strict flanking positions). The aligned 18S and 28S data sets contained 2299/1043 and 3695/1729 total nucleotide positions, respectively, before/after processing in Gblocks. We manually and unambiguously aligned EF-1α after removal of the intron in position 753/754 (present in most, but not all Coleoptera) and a few other taxon-specific introns. The fragment of EF-1α sequenced contained 1058 bp (excluding introns) corresponding to positions 118–1176 in the Drosophila F1 copy. The resulting alignments (6-gene, rDNA, & EF-1α) were concatenated in Mesquite 2.5 and the resulting supermatrix (12,778/9566 bp before/after processing of 18S and 28S in Gblocks) was used in subsequent analyses.

### DNA isolation, amplification & sequencing

Protocols used for genomic DNA isolation, amplification and sequencing are published elsewhere [55], [62], [63]. DNA sequencing was performed on ABI 3730 sequencers at the Harvard University Bauer Core Facilities. Sequences were assembled and edited with Sequencher 4.6. Specimen vouchers (for new sequences) have been deposited at the Harvard University Museum of Comparative Zoology, and nucleotide sequences newly determined here have been deposited in GenBank under accession nos. HM156701-HM156727.

### Phylogenetic analyses

Probabilistic model-based phylogenetic analyses were conducted on the 9-gene molecular supermatrix using Bayesian inference (BI) in the program MrBayes 3.1.2 [64], [65] and maximum likelihood (ML) inference in the program GARLI 0.951 [66]. We used ModelTest Server running ModelTest 3.8 [67], [68] and MrModeltest 2.3 for the statistical selection of models of nucleotide substitution (confidence level for LRT's  = 0.01 with branch lengths counted as parameters). Input files with likelihood scores for the set of candidate models were obtained from execution of ModelBlock and MrModelBlock files in PAUP* 4.0b10 [69].

Two paired Bayesian analyses (4 runs) were executed on the 9-gene data set ((partitions: AATS, CAD, SNF, PGD, RNA POL II, and EF-1α – 1^st^, 2^nd^, and 3^rd^ positions, 28S, 18S), GTR+I+G (model parameters partitioned by gene region), estimated base frequencies, 4 chains, trees sampled every 1000 generations). Both paired analyses converged (as measured by the standard deviation of split frequencies falling below .01) by 2.0×10^6^ generations, and were run for a total of 5.0×10^6^ generations. To further diagnose convergence and otherwise check performance and accuracy of the analyses, we implemented a series of graphical and statistical analyses on the resulting log files in the programs Tracer 1.4 and AWTY. Based on these analyses we imposed a conservative burn-in on each tree file and combined the last 1,000 trees from each of the 4 runs. We used the resulting 4,000 trees to estimate PP's and to obtain a 50% majority-rule consensus tree (using PAUP* 4.03b10 [69] and TreeAnnotator 1.4.7). Bayesian posterior probability (BPP) values ≥0.95 were considered to constitute strong nodal support.

Four tree searches (5×10^6^ generations) were implemented under maximum likelihood (ML) inference on the 9-gene supermatrix in the program GARLI 0.951 (GTR+I+G, estimated base frequencies). A ML bootstrap analysis was implemented in GARLI 0.96 (500 inferences, each terminated after 10,000 generations without improving topology) using Portal 2.2 to access the CIPRES cluster at the San Diego Supercomputing Center. Maximum likelihood bootstrap support (BS) values ≥90% were considered to constitute strong nodal support.

Strepsiptera exhibited unusually long branches in previous analyses, particularly those employing rDNA [e.g., 41]. While LBA can be problematic for parsimony analyses [50], it is generally less of a problem with probabilistic model-based approaches to phylogenetic inference such as BI and ML [70], [71]. Nonetheless, we conducted replicate BI and ML analyses on a 7-gene matrix, which was identical to the 9-gene matrix except for the exclusion of 18S and 28S, to see how the inclusion of rDNA sequence data affected the relationships recovered.

Parsimony analyses were conducted on the 9-gene and 7-gene matrices in the program PAUP* 4.03b10 [69]. Equally weighted heuristic tree searches were performed using the parsimony ratchet procedure [72] with 1000 replicates as implemented in the program PAUPRat on the CIPRES cluster. The resulting most parsimonious trees were used to start equal weights heuristic tree searches. Nodal support was evaluated with 1000 non-parametric bootstrap pseudoreplicates (10 RAS of taxa, TBR branch swapping).

## Results and Discussion

### Phylogeny of Holometabola

**Figure 2:**
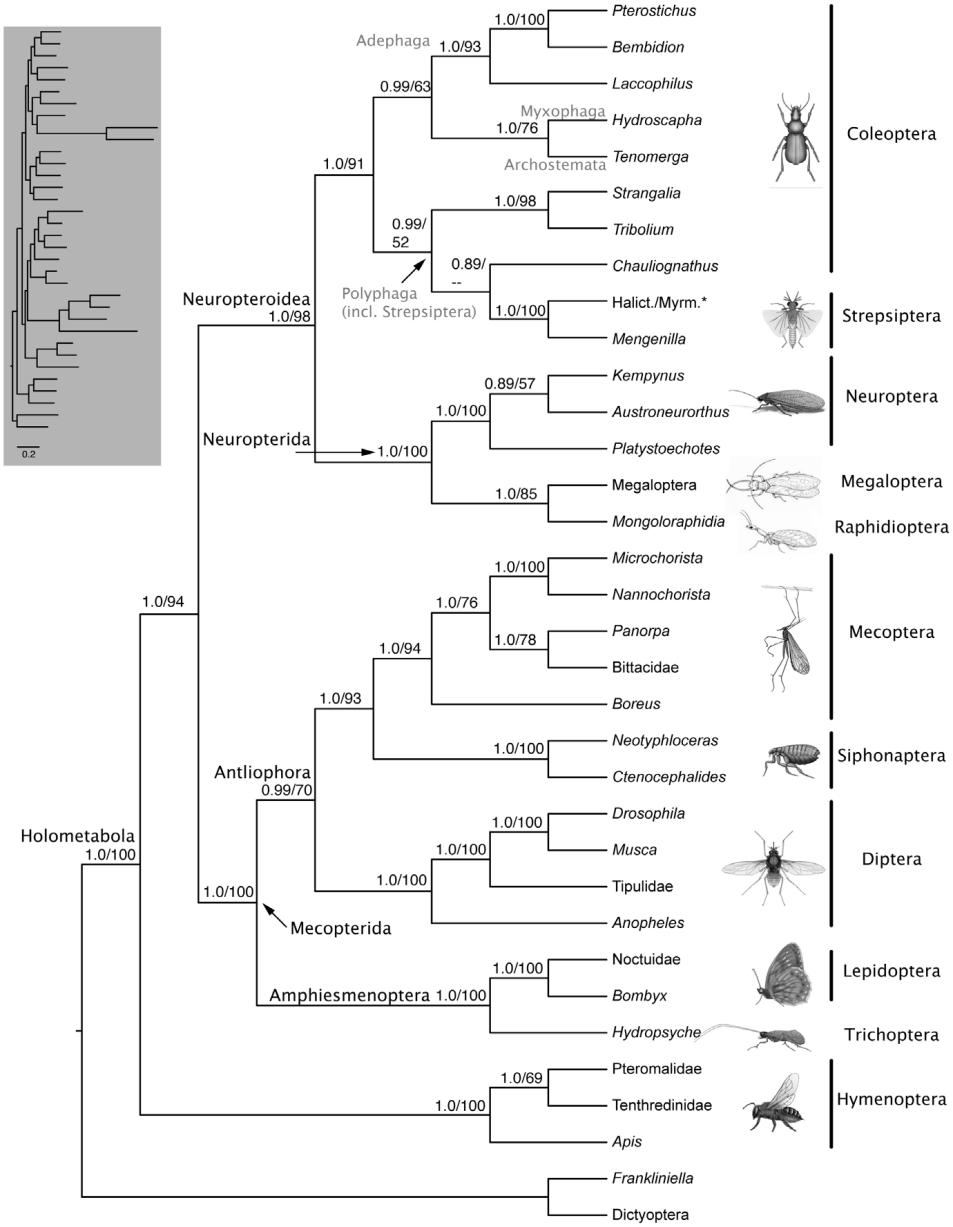
Congruent Bayesian/ML phylogenetic tree showing the placement of Strepsiptera in Coleoptera and interrelationships of other holometabolous insects based on the combined analysis of DNA sequence data from 9 genes. Bayesian PP's ≥0.50 and ML BS values ≥50% are shown above branches (BPP/BS). Note the relatively long branches subtending Diptera and Strepsiptera (see inset), consistent with previous studies [e.g., 28,29,40]. Images of insect exemplars are not to scale. Images of Raphidioptera and Megaloptera copyright Ainsley Seago, other insect images copyright Australian Museum. * Halictophagidae/Myrmecolacidae

BI and ML analyses of the 9-gene matrix recovered congruent phylogenetic trees (Fig. 2) with strong support for monophyly of the holometabolous insect orders, excluding the placement of Strepsiptera (itself monophyletic) in Coleoptera (0.89 BPP/<50% BS). Hymenoptera was sister to all other Holometabola, consistent with other recent studies [28]–[30], [73]–[77]. Two major lineages were recovered within Holometabola: Mecopterida (also known as Panorpida) (1.0/100%), comprised of Diptera, Lepidoptera, Mecoptera, Siphonaptera and Trichoptera, and Neuropteroidea (1.0/98%;  =  Neuropterida + Coleoptera) [*sensu* 29], comprised of Coleoptera, Neuropterida (1.0/100%; comprised of Megaloptera, Neuroptera and Raphidioptera) and Strepsiptera. Both groupings are in accordance with most recent molecular and morphological hypotheses [28], [29], [44], [46]. Within Mecopterida we recovered the supraordinal groupings Amphiesmenoptera (1.0/100%), comprised of Lepidoptera and Trichoptera, and Antliophora (1.0/70%), comprised of Diptera, Mecoptera and Siphonaptera. These were the same ordinal- and supra-ordinal groupings recovered by Wiegmann et al. [28], [29], apart from the placement of Strepsiptera.

We recovered improved BS and/or BPP support compared to Wiegmann et al. [29] for Neuropteroidea (98% vs. 89% BS), Neuropterida (91% vs. 83% BS), Mecopterida (100% vs. 66% BS), and for the sister group relationship between Siphonaptera and Mecoptera (1.0 BPP/93% BS vs. 0.81/50%); however, we recovered relatively lower BS support for Antliophora (70% vs. 86%). Lower-level topological differences between our results and those of Wiegmann et al. [29] included the placement of *Platystoechotes* sister to the remaining Neuroptera in the 9-gene trees and in BI analyses of the 7-gene matrix (*Platystoechotes* was sister to *Kempynus* in ML analyses of the 7-gene matrix, consistent with Wiegmann et al. [29]), the placement of Bittacidae sister to *Panorpa* in the 9-gene trees (Bittacidae was sister to the remaining Mecoptera in the 7-gene trees, consistent with Wiegmann et al. [29]), and in the 7-gene trees the placement of *Anopheles* sister to a clade comprised of *Drosophila* and *Musca* under ML inference and sister to *Drosophila* under BI (*Anopheles* was sister to the remaining Diptera in the 9-gene trees, consistent with Wiegmann et al. [29]). Relationships recovered by Longhorn et al. [30] differed somewhat depending on the method of character coding and analysis, and internodal BPP and BS support was mostly lower than in our trees.

The 9-gene parsimony tree (Fig. S1) notably failed to recover the supraordinal groupings Mecopterida, Neuropteroidea and Antliophora, and recovered a paraphyletic order Coleoptera. The 7-gene parsimony tree (Fig. S2) failed to recover the supraordinal groupings Mecopterida, Neuropterida and Antliophora, and recovered a polyphyletic order Coleoptera. Diptera was rendered paraphyletic in the 7-gene parsimony tree by the peculiar placement of *Tribolium* sister to a clade comprised of *Anopheles* and *Drosophila*. Both the 9-gene and 7-gene trees recovered Siphonaptera in a position sister to Mecoptera (84% 9-gene/54% 7-gene).

### Phylogenetic placement of Strepsiptera

**Figure 3:**
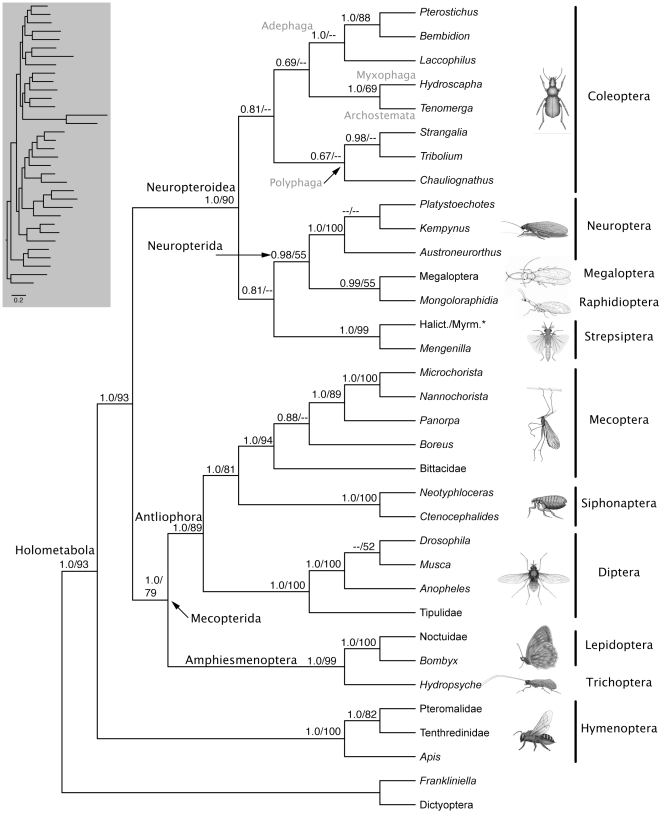
ML phylogenetic tree showing the placement of Strepsiptera sister to Neuropterida and interrelationships of other holometabolous insects based on the combined analysis of DNA sequence data from 7 single-copy nuclear protein-coding genes. Bayesian posterior probabilities ≥0.50 and ML BS values ≥50% are shown above branches (BPP/BS). Images of insect exemplars are not to scale. Images of Raphidioptera and Megaloptera copyright Ainsley Seago, other insect images copyright Australian Museum. * Halictophagidae/Myrmecolacidae

We recovered Strepsiptera within Coleoptera in the suborder Polyphaga sister to *Chauliognathus* (Elateroidea: Cantharidae) when rDNA were included in the BI and ML analyses (9-gene matrix; Fig. 2), or sister to Neuropterida when rDNA were excluded and the BI and ML analyses were limited to a matrix composed of the seven single-copy nuclear protein-coding genes (Fig. 3). Strepsiptera were recovered sister to Diptera (84%) in parsimony analyses of the 9-gene matrix (Fig. S1), and in a position sister to the beetle *Chauliognathus* (66%) within Neuropteroidea (minus *Tribolium*) in parsimony analyses of the 7-gene matrix (Fig. S2). However, Strepsiptera exhibited unusually long branches in previous analyses, and their placement in parsimony analyses is expected to result at least in part from systematic bias introduced by LBA [see 41]. This may be especially true for the 9-gene parsimony tree on account of the inclusion of rDNA sequence data.

None of our analyses recovered Strepsiptera in a position sister to Coleoptera. Nonetheless, we recovered low/limited nodal support under BI and ML inference for the placement of Strepsiptera within Coleoptera (0.89/<50%), or as the sister group to Neuropterida (0.81/<50%), and the subordinal relationships we recovered within Coleoptera (Figs. 2,3) differed from other recent molecular phylogenetic studies [e.g., 54,55] in the placement of Adephaga sister to a clade comprised of exemplars from the suborders Myxophaga and Archostemata (1.0/63%; Fig. 2); however, relationships within Coleoptera were otherwise consistent with other recent studies [e.g., 54,55]. Placement of Strepsiptera within the beetle suborder Polyphaga when rDNA were included in the analysis (9-gene trees) is nonetheless intriguing. The inclusion of rDNA resulted in the same or higher BPP and BS support for equivalent internodes when compared to analyses lacking rDNA sequence data (7-gene analyses) with just two exceptions: (1) Antliophora (70% BS 9-gene, 89% BS 7-gene tree), and (2) the internode uniting Pteromalidae and Tenthredinidae (69% BS 9-gene, 82% BS 7-gene).

Note that when all Coleoptera except *Strangalia* and *Tribolium* (the only Coleoptera included in Wiegmann et al. [28], [29]) are pruned from our 9-gene tree (Fig. 2), Strepsiptera are sister to Coleoptera, consistent with the results of Wiegmann et al. [28], [29]. Therefore, while the sister group relationship between Strepsiptera and Coleoptera recovered by Wiegmann et al. [29] is compatible with results from analyses of our 9-gene supermatrix (with a comprehensive sample of coleopteran suborders), interpretation of the results of Wiegmann et al. [29] as demonstrating a sister group relationship between Strepsiptera and Coleoptera may be an artifact of taxon sampling. Wiegmann et al. [29] sampled only two beetles (*Strangalia* and *Tribolium*), both of which belong to the suborder Polyphaga [54], [55]. The other three extant suborders of Coleoptera (Adephaga, Archostema and Myxophaga) were not sampled. The results of Longhorn et al. [30], while generally in support of a close (perhaps even sister group) relationship between Strepsiptera and Coleoptera, are difficult to interpret on account of incomplete taxon sampling at the ordinal level within Holometabola and at the subordinal level within Coleoptera (lacking Archostemata), and overall lesser well-supported resolution.

Note that none of our analyses recovered evidence for a close relationship between Strepsiptera and any other group of holometabolous insects outside of Neuropteroidea. It is further worth noting that the presence of an intron in position 753/754 in the EF1-α gene of all Strepsiptera examined (Myrmecolacidae HM156724, EF588666; Halictophagidae EF666135; Mengenillidae EF666133; Tridactylophagidae EF666137) and in most other members of the class Insecta, is inconsistent with the concept of Halteria; loss of this intron is an apparent synapomorphy for Mecopterida, in which Halteria would reside. This observation is consistent with studies of an engrailed homeobox intron [47] and of ecdysone receptor and ultraspiracle proteins [51], [52], which also contradict a close relationship between Diptera and Strepsiptera.

On account of the incomplete sampling of coleopteran suborders by Wiegmann et al. [29] and Longhorn et al. [30], exclusion of Neuropterida and Mecoptera from the taxon sample of Longhorn et al. [30], missing DNA sequence data and consequent extensive white space in Wiegmann et al. [29], Longhorn et al. [30], and the present study, and the known problems with LBA/evolutionary rates and alignment of rDNA (relevant to the placement of Strepsiptera within Coleoptera in the present 9-gene study, but presumably ameliorated by the methods of analysis employed), we propose that at least three viable alternative hypotheses remain for the phylogenetic placement of Strepsiptera (in random order): (a) as sister group to Coleoptera [e.g., 23,28–30,32,33], (b) as sister group to Neuropterida (the present 7-gene data set), or (c) within Coleoptera (the present 9-gene data set [e.g., 20,21,31]), most likely derived from within the suborder Polyphaga (the present 9-gene data set [21]). Thus, while the phylogenetic neighborhood of Strepsiptera has been identified, unequivocal placement to a specific branch within Neuropteroidea will require additional study.

## Supporting Information

Figure S1 Single most parsimonious tree showing the placement of Strepsiptera sister to Diptera and interrelationships of other holometabolous insects based on the combined analysis of DNA sequence data from 9 genes. Parsimony bootstrap support ≥50% is shown above branches. * Halictophagidae/Myrmecolacidae
(6.17 MB TIF)

Figure S2 Single most parsimonious tree showing the placement of Strepsiptera within Neuropteroidea and interrelationships of other holometabolous insects based on the combined analysis of DNA sequence data from 7 genes (no rDNA). Parsimony bootstrap support ≥50% is shown above branches. * Halictophagidae/Myrmecolacidae
(6.38 MB TIF)

Table S1 Taxa and genes sampled.
(0.08 MB DOC)

Table S2 Primers used for amplification and sequencing.
(0.03 MB DOC)
