# Supplementary figures and images for: 9-Genes Reinforce the Phylogeny of Holometabola and Yield Alternate Views on the Phylogenetic Placement of Strepsiptera
Source: PLoS One. 2010 Jul 29;5(7):e11887. doi: 10.1371/journal.pone.0011887 (PMC2912379; doi:10.1371/journal.pone.0011887)

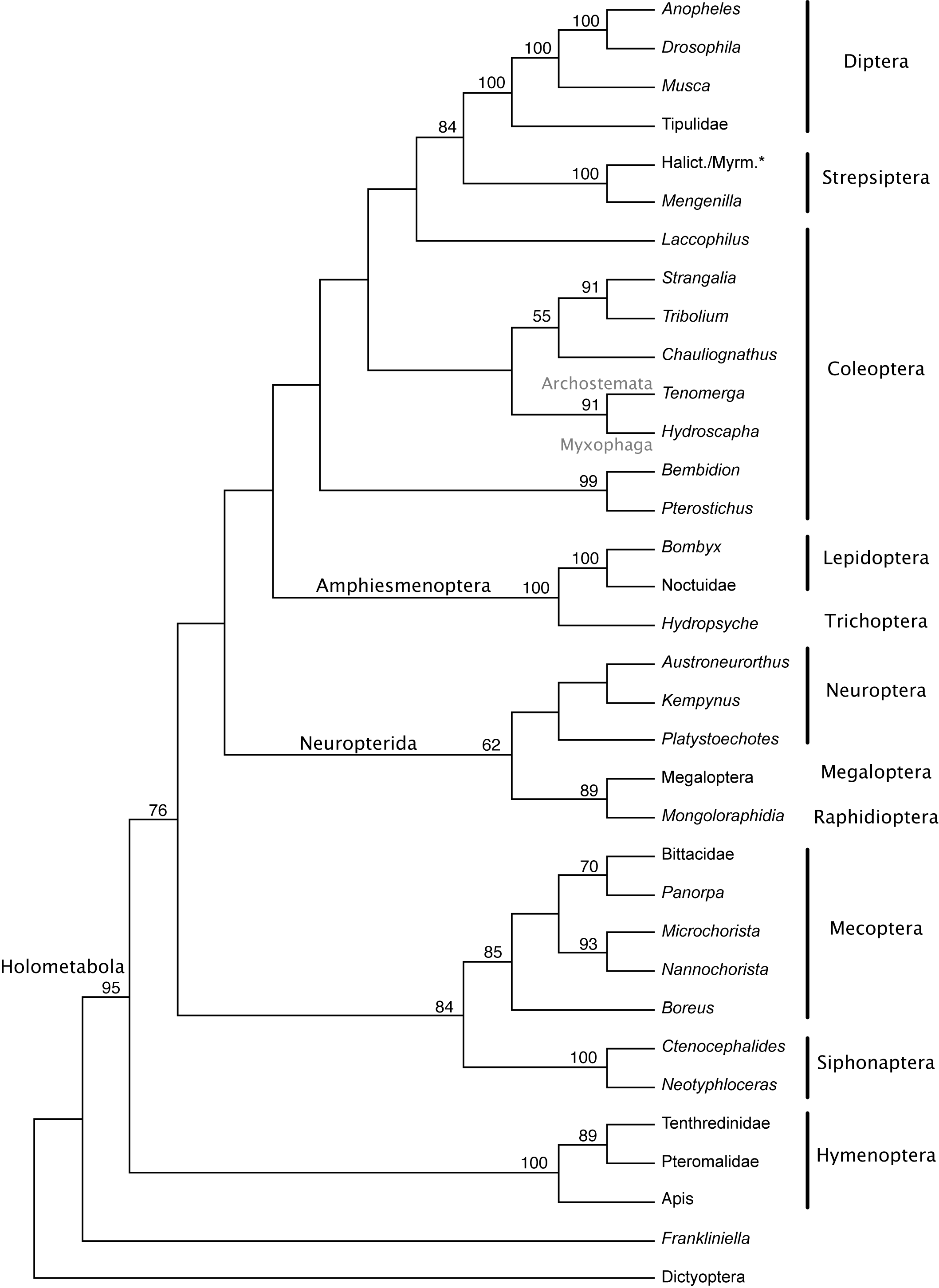

Supplement: Figure S1 — Single most parsimonious tree showing the placement of Strepsiptera sister to Diptera and interrelationships of other holometabolous insects based on the combined analysis of DNA sequence data from 9 genes. Parsimony bootstrap support ≥50% is shown above branches. * Halictophagidae/Myrmecolacidae (6.17 MB TIF) [file pone.0011887.s001.tif]

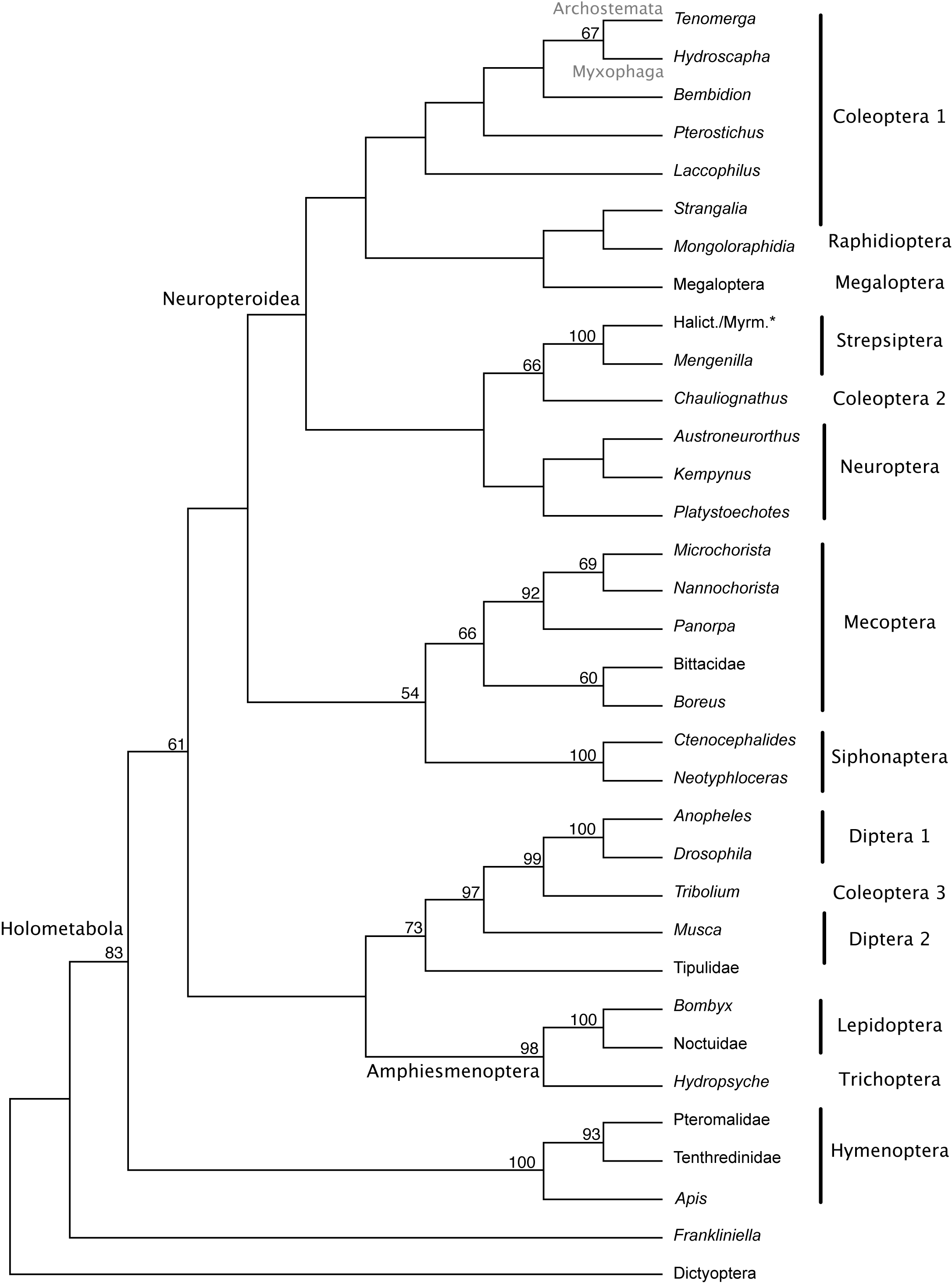

Supplement: Figure S2 — Single most parsimonious tree showing the placement of Strepsiptera within Neuropteroidea and interrelationships of other holometabolous insects based on the combined analysis of DNA sequence data from 7 genes (no rDNA). Parsimony bootstrap support ≥50% is shown above branches. * Halictophagidae/Myrmecolacidae (6.38 MB TIF) [file pone.0011887.s002.tif]
