# Supplementary material for: 9-Genes Reinforce the Phylogeny of Holometabola and Yield Alternate Views on the Phylogenetic Placement of Strepsiptera
Source: PLoS One. 2010 Jul 29;5(7):e11887. doi: 10.1371/journal.pone.0011887 (PMC2912379; doi:10.1371/journal.pone.0011887)
Supplement: Table S1 — Taxa and genes sampled. (0.08 MB DOC) [file pone.0011887.s003.doc]

| **ORDER: Suborder (Coleoptera only)** | **Family** | **Genus/species** | **Gene** | **GenBank Accession No.** |
| --- | --- | --- | --- | --- |
| COLEOPTERA |  |  |  |  |
| Adephaga | Carabidae | *Bembidion louisella* | 18S | EF648670 |
| Adephaga | Carabidae | *B. perspicuum* | EF-1α, 28S | GQ503346, GQ503347 |
| Adephaga | Carabidae | *B. transversale* | RNA Pol II, CAD | EU677589, EU677541 |
| Adephaga | Carabidae | *Pterostichus* | EF-1α, 28S | HM156716, HM156706 |
| Adephaga | Carabidae | *P. globosus* | 18S | FJ173132 |
| Adephaga | Carabidae | *P. melanarius* | RNA Pol II, CAD | EU677582, EU677533 |
| Adephaga | Dytiscidae | *Laccophilus* | EF-1α, 28S | HM156717, HM156707 |
| Adephaga | Dytiscidae | *L. poecilus* | 18S | AJ318714 |
| Adephaga | Dytiscidae | *L. pictus* | RNA Pol II, CAD | EU677586, EU677528 |
| Archostemata | Cupedidae | *Tenomerga cinerea* | EF-1α, 28S, 18S, RNA Pol II, CAD | GQ503344, EU797392, EU797417, EU677579, EU677525 |
| Myxophaga | Hydroscaphidae | *Hydroscapha natans* | EF-1α, 28S, 18S, RNA Pol II, CAD | HM156718, HM156708, AF012525, HM156727, HM156726 |
| Polyphaga | Cerambycidae | *Strangalia bicolor* | AATS, CAD, RNA Pol II | GQ265574, GQ265599, GQ265664 |
| Polyphaga | Cerambycidae | *S. luteicornis* | EF-1α, 28S, 18S | HM156719, HM156701, HM156709 |
| Polyphaga | Cantharidae | *Chauliognathus opaca* | EF-1α, 18S, 28S, RNA Pol II, CAD | HM156720, HM156710, HM156702, EU677580, EU677598 |
| Polyphaga | Tenebrionidae | *Tribolium* | 18S, 28S, EF-1α, AATS, CAD, PGD, SNF, TPI, RNA Pol II | HM156711, HM156703, HM156722, XM_970534, EU677538, XM_966958, XM_963178, XM_970400, XM_968377 |
| DIPTERA |  |  |  |  |
|  | Tipulidae | *Tipula* | 18S | X89496 |
|  | Tipulidae | *T. abdominalis* | 28S, AATS, CAD, PGD, SNF | FJ040553, GQ265563, GQ265584 GQ265611, GQ265626 |
|  | Tipulidae | *Holorusia rubiginosa* | EF-1α | AF423809 |
|  | Culicidae | *A. annulipes* | EF-1α | DQ420615 |
|  | Culicidae | *A. gambiae* | 18S, 28S, AATS, CAD, PGD, SNF, TPI, RNA Pol II | AM157179, AF417813, XM_318757, XM_310823, XM_313091, XM_320869, XM_321467, XM_313929 |
|  | Muscidae | *Musca domestica* | 18S, 28S, EF-1α, AATS, CAD, PGD, SNF, TPI | GQ465780, AY123358, GQ465788, GQ265564, GQ265585 GQ265612, GQ265627 GQ265639 |
|  | Drosophilidae | *Drosophila melanogaster* | 18S, 28S, EF-1α, AATS, CAD, PGD, SNF, TPI, RNA Pol II | M21017, M21017, X06869, NM_205934, X04813, M80598, NM_078490, NM_176587, NM_078569 |
| HYMENOPTERA |  |  |  |  |
|  | Apidae | *Apis mellifera* | EF-1α, 28S, 18S, AATS, CAD, PGD, SNF, TPI, RNA Pol II | AF015267, AY703551, AY703484, XM_395392, XM_393888, XM_625087, XM_393440, XR_014889, XM_623278 |
|  | Tenthredinidae | *Ametastegia equiseti* | AATS, CAD, PGD, SNF, TPI, RNA Pol II | GQ265565, GQ265586 GQ265587, GQ265613 GQ265628, GQ265640 GQ265656 |
|  | Tenthredinidae | *Athalia rosea* | 18S, 28S, EF-1α | AB064266, AB064267, AB253792 |
|  | Pteromalidae | *Muscidifurax raptorellus* | AATS, CAD, PGD, SNF, TPI, RNA Pol II | GQ265578, GQ265604 GQ265605, GQ265606 GQ265624, GQ265634 GQ265650, GQ265668 |
|  | Pteromalidae | *M. raptor* | 28S | AY855207 |
|  | Pteromalidae | *Mesopolobus* sp. | 18S | AY949825 |
|  | Pteromalidae | *Quadrasticus erythrina* | EF-1α | FJ949570 |
| LEPIDOPTERA |  |  |  |  |
|  | Saturniidae | *Bombyx mori* | 28S, EF-1α, 18S, AATS, CAD, PGD, SNF, TPI | HM156712, HM156723, DQ347470, M55993, EU032656, NM_001047060, DQ202313, NM_001126258 |
|  | Noctuidae | *Helicoverpa armigera* | 28S | GU350477 |
|  | Noctuidae | *H. assulta* | 18S | EU057177 |
|  | Noctuidae | *Heliothis virescens* | EF-1α, AATS, CAD, PGD, TPI, RNA Pol II | U20135, GQ265570, GQ265592, GQ265618, GQ265644, GQ265660 |
| MECOPTERA |  |  |  |  |
|  | Nannochoristidae | *Microchorista philpotti* | 28S, AATS, CAD, PGD, TPI, RNA Pol II | FJ040545, GQ265560, FJ040617, GQ265608, GQ265635, GQ265652 |
|  | Nannochoristidae | *Nannochorista* sp. | AATS, CAD, PGD, SNF, TPI, RNA Pol II | GQ265571, GQ265593/GQ265594, GQ265619 GQ265631, GQ265645, GQ265661 |
|  | Nannochoristidae | *N. neotropica* | 18S, 28S, EF-1α | AF334799, AF338261, AF423848 |
|  | Bittacidae | *Australobittacus* sp. | AATS, CAD, PGD, RNA Pol II | GQ265577, GQ265602, GQ265603, GQ265667 |
|  | Bittacidae | *Apterobittacus apterus* | 18S, 28S, EF-1α | AF423875, AF423926, AF423817 |
|  | Boreidae | *Boreus brumalis* | 18S, 28S, EF-1α, AATS, CAD, RNA Pol II | AF423883, AF423936, AF423828, GQ265576, GQ265601, GQ265666 |
|  | Panorpidae | *Panorpa* sp. | AATS, CAD, PGD, SNF, TPI, RNA Pol II | GQ265572, GQ265595 GQ265620, GQ265632 GQ265646, GQ265662 |
|  | Panorpidae | *P. cognata* | 18S, 28S, EF-1α | AF423897, AF423954, AF423851 |
| MEGALOPTERA |  |  |  |  |
|  | Corydalidae | *Nigronia* sp. | CAD, PGD, TPI | GQ265598, GQ265623, GQ265648 |
|  | Corydalidae | *N. serricornis* | 18S | EU815263 |
|  | Sialidae | *Sialis hamata* | 28S, EF-1α | HM156713, HM156721 |
| NEUROPTERA |  |  |  |  |
|  | Neurorthidae | *Austroneurorthus brunneipennis* | 18S, AATS, CAD, TPI, RNA Pol II | EU815229, GQ265575, GQ265600, GQ265649, GQ265665 |
|  | Osmylidae | *Kempynus* sp. | 18S, AATS, CAD, PGD | EU815249, GQ265567, GQ265589 GQ265615 |
|  | Polystoechotidae | *Platystoechotes* sp. | 18S, AATS, CAD, PGD, SNF, TPI, RNA Pol II | EU815274, GQ265568, GQ265590 GQ265616, GQ265629, GQ265642, GQ265658 |
| RAPHIDIOPTERA |  |  |  |  |
|  | Raphidiidae | *Mongoloraphidia martrynovae* | 18S, CAD, PGD | EU815252, GQ265597 GQ265622 |
|  | Raphidiidae | *M. manasiana* | EF-1α | AY620204 |
| SIPHONAPTERA |  |  |  |  |
|  | Pulicidae | *Ctenocephalides canis* | 18S, 28S, EF-1α | AF423914, AF423974, AF423870 |
|  | Pulicidae | *C. felis* | AATS, CAD, PGD, SNF, TPI, RNA Pol II | GQ265561, GQ265581 GQ265609, GQ265625 GQ265636, GQ265653 |
|  | Hystrichopsyllidae | *N. crassipina chilensis* | 18S, 28S, EF-1α | EU336079, EU336187, EU336293 |
|  | Hystrichopsyllidae | *Neotyphloceras* sp. | AATS, CAD, RNA Pol II | GQ265579, GQ265607, GQ265669 |
| STREPSIPTERA |  |  |  |  |
|  | Myrmecolacidae | *Caenocholax* | 28S, EF-1α, 18S | HM156704, HM156724, HM156714 |
|  | Halictophagidae | Gen. sp. | AATS, CAD, PGD, TPI, RNA Pol II | GQ265562, GQ265583 GQ265610, GQ265638, GQ265655 |
|  | Mengenillidae | *Mengenilla* | 18S, 28S, EF-1α, CAD, RNA Pol II | HM156715, HM156705, HM156725/EF666133, GQ265580, GQ265651 |
| TRICHOPTERA |  |  |  |  |
|  | Hydropsychidae | *Hydropsyche* | 18S, 28S, EF-1α | AF286291, AF338267, AF436621 |
|  | Hydropsychidae | *H. phalerata* | AATS, CAD, PGD, SNF, TPI, RNA Pol II | GQ265569, GQ265591 GQ265617, GQ265630 GQ265643, GQ265659 |
| DICTYOPTERA (OUTGROUP) |  |  |  |  |
|  | Blattellidae | *Blatella germanica* | 18S, 28S, AATS, CAD, PGD, SNF, TPI, RNA Pol II | AF005243, AF005243, GQ265573, GQ265596 GQ265621, GQ265633 GQ265647, GQ265663 |
|  | Blattidae | *Periplaneta americana* | EF-1α | AY305517 |
| THYSANOPTERA (OUTGROUP) |  |  |  |  |
|  | Thripidae | *Frankliniella* sp. KY-2004 | 18S | AY630445 |
|  | Thripidae | *F. occidentalis* | 28S, EF-1α | AY523391, GU148015 |
|  | Thripidae | *F. fusca* | AATS, CAD, PGD, TPI, RNA Pol II | GQ265566, GQ265588 GQ265614, GQ265641, GQ265657 |
