# Supplementary material for: 9-Genes Reinforce the Phylogeny of Holometabola and Yield Alternate Views on the Phylogenetic Placement of Strepsiptera
Source: PLoS One. 2010 Jul 29;5(7):e11887. doi: 10.1371/journal.pone.0011887 (PMC2912379; doi:10.1371/journal.pone.0011887)
Supplement: Table S2 — Primers used for amplification and sequencing. (0.03 MB DOC) [file pone.0011887.s004.doc]

| **Locus** | **Name** | **Use** | **Sequence** (5’→3') | **Citation** |
| --- | --- | --- | --- | --- |
| 18S | 18e-s | Amp/Seq | ctggttgatcctgccagt | 1 |
| 18S | 18p-c | Amp/Seq | taatgatccttccgcaggttcacct | 1 |
| 18S | 18S1.2f | Seq | TGCTTGTCTCAAAGATTAAGC | 2 |
| 18S | r1138 | Amp/Seq | GTTAGAGGTTCGAAGGCG | 3 |
| 18S | f1094 | Amp/Seq | GGATCGTCGCAAGACGGACAGAAG | 3 |
| 28S | rD4.2b | Amp/Seq | CCTTGGTCCGTGTTTCAAGACGG | 2 |
| 28S | rD5b | Amp/Seq | CCACAGCGCCAGTTCTGCTTAC | 2 |
| 28S | ZX1 | Amp/Seq | ACCCGCTGAATTTAAGCATAT | 4 |
| 28S | OP2 | Amp/Seq | cagactagagtcaagctcaacagg | 5 |
| 28S | ZR1 | Seq | gtcttgaaacacggaccaaggagtct | 5 |
| EF-1α | Cho10(mod) | Amp | ACRGCVACKGTYTGHCKCATGTC | 6 |
| EF-1α | Cho10rev1 | Amp/Seq | AGCATCDCCAGAYTTGATRGC | 7 |
| EF-1α | efa747 | Amp/Seq | CCACCAATTTTGTAGACATC | 8 |
| EF-1α | efs372 | Amp/Seq | CTGGTGAATTTGAAGCYGGTA | 9 |
| EF-1α | for1deg | Amp/Seq | GYATCGACAARCGTACSATYG | 6 |

# Literature Cited for Table S2

1. Halaynch K, Lutz RA, Vrijenhoek RC (1998) Evolutionary origins and age of vestimentiferan tube-worms. Cah Biol Mar 39: 355–358.
2. Whiting MF (2002b) Mecoptera is paraphyletic: multiple genes and phylogeny of Mecoptera and Siphonaptera. Zool Scr 31: 93–104.
3. Sequeira AS, Normark BB, Farrell BD (2000) Evolutionary assembly of the conifer fauna: Distinguishing ancient from recent associations in bark beetles. Proc R Soc Lond B Biol Sci 267: 2359–2366.
4. Van der Auwera G, Chapelle S, De Wachter R (1994) Structure of the large ribosomal subunit RNA of Phytophthora megasperma, and phylogeny of the Oomycetes. FEBS Lett 338: 133–136.
5. Mallatt J, Sullivan J (1998) 28S and 18S ribosomal DNA sequences support the monophyly of lampreys and hagfishes. Mol Biol Evol 15: 1706–1718.
6. Danforth BN, Ji S (1998) Elongation factor–1alpha occurs as two copies in bees: Implications for phylogenetic analysis of EF–1alpha sequences in insects. Mol Biol Evol 15: 225–235.
7. McKenna DD, Sequeira AS, Marvaldi AE, Farrell BD (2009) Temporal lags and overlap in the diversification of weevils and flowering plant. Proc Natl Acad Sci USA 106: 7083-7088.
8. Normark BB, Jordal BH, Farrell BD (1999) Origin of a haplodiploid beetle lineage. Proc R Soc Lond B Biol Sci 266: 2253–2259.
9. McKenna DD, Farrell BD (2005) Molecular phylogenetics and evolution of host plant use in the Neotropical rolled leaf 'hispine' beetle genus Cephaloleia (Chevrolat) (Chrysomelidae: Cassidinae). Mol Phylogenet Evol 37: 117–131.
